# A59 GLOBAL PROTEOMIC PROFILING OF HUMAN COLONOID MONOLAYERS UNDERGOING IN VITRO CHRONIC DAMAGE

**DOI:** 10.1093/jcag/gwac036.059

**Published:** 2023-03-07

**Authors:** S Sandilya, T Steiner, P Lange, W Rees

**Affiliations:** 1 Department of Microbiology and Immunology; 2 Department of Medicine; 3 Department of Pathology and Laboratory Medicine; 4 Experimental Medicine, University of British Columbia, Vancouver, Canada; 5 scientist, T Rex Bio, San Francisco, United States

## Abstract

**Background:**

An *in vitro* damage model has been established in our lab using human colonoids grown as 2D monolayers. Upon being subjected to repeated rounds of air-liquid interface (ALI) growth and injury by submergence, these colonoid monolayers lost their barrier integrity and regrowth potential. Changes in mRNA expression and DNA methylation in genes from this human model of injury were similar to those that occur in Inflammatory Bowel Disease (IBD) and colon cancer. Significant morphological changes were observed in these monolayers after they were subjected to subsequent rounds of submergence injury, compared to when they were differentiated in ALI.

**Purpose:**

Submergence injury is predicted to be involved in unfolded protein response (UPR) activation which can specifically alter translation. Hence proteomics studies will help undertand these changes.

**Method:**

To determine if these changes are mirrored in the proteomes of damaged colonoids, we employed a Single-Plot, Solid-Phase-enhanced Sample Preparation (SP3) technology for Mass Spectrometry (MS) based proteomics analysis to characterize these monolayers at baseline, once they were differentiated in ALI, after one and five rounds of injury after differentiation in ALI, and after stimulation with the Toll-like receptor 5 (TLR5) agonist FliC. Hierarchical clustering, enrichment analysis, volcano plot analysis after pre-processing and normalization of the proteomics data set revealed differentially expressed proteins across various groups of monolayers.

**Result(s):**

Preliminary proteomic data analysis revealed changes in the profile of proteins involved in cellular differentiation, mitochondrial proteins, hypoxia upregulated proteins, those responsible for the maintenance and reorganization of the cytoskeletal structure and Golgi structure. These changes in protein profile may account for the significant morphological changes observed in these monolayers when subjected to submergence injury. Some outliers in monolayers subjected to microbial stimulation included proteins involved in regulation of extracellular matrix dependent motility and components of Adaptor Protein Complexes. Further studies are needed to ascertain if these account for the protective effect of FliC on these monolayers.

**Conclusion(s):**

This study suggests that the submergence injury to these healthy human derived colonoid monolayers leads to changes in their protein profile which mirror those seen in case of acute and chronic inflammation like IBD and colon cancer. It corroborates with the findings of gene expression and epigenetic analyses using the *in vitro* model established in our lab.

**Please acknowledge all funding agencies by checking the applicable boxes below:**

CCC

**Disclosure of Interest:**

None Declared

